# Screening mammography: benefit of double reading by breast density

**DOI:** 10.1007/s10549-018-4864-1

**Published:** 2018-07-04

**Authors:** My von Euler-Chelpin, Martin Lillholm, George Napolitano, Ilse Vejborg, Mads Nielsen, Elsebeth Lynge

**Affiliations:** 1 Department of Public Health, University of Copenhagen, Øster Farimagsgade 5, 1014 Copenhagen K, Denmark; 2 Biomediq, Fruebjergvej 3, 2100 Copenhagen Ø, Denmark; 3 Department of Radiology, University Hospital Copenhagen Rigshospitalet, Blegdamsvej 9, 2100 Copenhagen Ø, Denmark; 4 Department of Computer Sciences, University of Copenhagen, Universitetsparken 5, 2100 Copenhagen Ø, Denmark

**Keywords:** Mammography, Screening, Single reading, Double reading, Sensitivity, Specificity

## Abstract

**Purpose:**

The currently recommended double reading of all screening mammography examinations is an economic burden for screening programs. The sensitivity of screening is higher for women with low breast density than for women with high density. One may therefore ask whether single reading could replace double reading at least for women with low density. We addressed this question using data from a screening program where the radiologists coded their readings independently.

**Methods:**

Data include all screening mammography examinations in the Capital Region of Denmark from 1 November 2012 to 31 December 2013. Outcome of screening was assessed by linkage to the Danish Pathology Register. We calculated sensitivity, specificity, number of interval cancers, and false positive-tests per 1000 screened women by both single reader and consensus BI-RADS density code.

**Results:**

In total 54,808 women were included. The overall sensitivity of double reading was 72%, specificity was 97.6%, 3 women per 1000 screened experienced an interval cancer, and 24 a false-positive test. Across all BI-RADS density codes, single reading consistently decreased sensitivity as compared with consensus reading. The same was true for specificity, apart from results across BI-RADS density codes set by reader 2.

**Conclusions:**

Single reading decreased sensitivity as compared with double reading across all BI-RADS density codes. This included results based on consensus BI-RADS density codes. This means that replacement of double with single reading would have negative consequences for the screened women, even if density could be assessed automatically calibrated to the usual consensus level.

## Background

The European Guidelines for quality assurance in breast cancer screening and diagnosis [1] recommend that a mammogram is read independently by two radiologists; also called double reading. According to the Guidelines, double reading enhances the sensitivity of the screening test with 5–15%, and sensitivity is certainly important to a screening program as it measures the ability of the screening test to find the cancers. Both the risk of breast cancer and the sensitivity of the screening test furthermore depend on the density of the breast tissue [2]. Breast density is often reported in four categories according to a system developed by American College of Radiology called Breast Imaging-Reporting and Data System (BI-RADS) [3].

In the population-based screening program of the Capital Region of Denmark, data have been collected on the outcome of the mammogram reading for each radiologist separately. This included both the BI-RADS density code and the categorization of the screening mammogram as negative or positive of malignancy. Women with negative mammography examinations were returned to routine screening, and women with positive mammography examinations were followed up with triple diagnostics.

European Guidelines require that a least one of the radiologist performing double reading of screening mammography examinations reads at least 5000 mammography examinations per year [1]. The limited number of qualified screening radiologists is a challenge, and double reading is a financial burden for the screening programs. On this basis, one may ask whether double reading of all mammography examinations is needed. Therefore, we took advantage of the BI-RADS density coded data from the Capital Region of Denmark to investigate the impact on the sensitivity and specificity of double versus single reading of mammography examinations stratified by level of breast density.

## Methods

### Screening

The Capital Region of Denmark offers biennial screening to women aged 50–69 years. Women are personally invited to visit one of the 5 mammography screening clinics in the region. The program uses the Siemens Inspiration digital mammography equipment. At screening, the radiographer takes a craniocaudal and an oblique view.

All mammography examinations are read and coded independently by two trained radiologists. If the two readers agree, the consensus code is their common code. If the two readers disagree on the malignancy code, a consensus code is made in dialog between the two readers, and if necessary a third independent reader is brought in. If the two readers disagree on the BI-RADS density code, the highest code is used as the consensus code. Normally, junior readers are first readers, but a given reader can advance to become second reader after some experience. So within the program, a given reader can therefore have acted in both roles.

In our dataset, breast density has been coded according to the 2003, 4th Edition of the BI-RADS density code [3]. BI-RADS 1 is fatty; where the breast is almost entirely fat (< 25% fibroglandular tissue); BI-RADS 2 is scattered (> 25–50%) fibroglandular; BI-RADS 3 is heterogeneously (51%-75%) dense; and BI-RADS 4 is dense (> 75%).

### Study base

We retrieved data on all screening mammography examinations from 1 November 2012 to 31 December 2013. Within the study period, no woman was screened more than once. The mammography register holds information on screening date, the outcome of each independent reading (including negative/positive code and BI-RADS density code), and the consensus outcome.

The outcome of screening was assessed by linkage to the Danish Pathology Register based on unique personal identification numbers used in both the screening register and in the pathology register. Women with a positive screening test and breast cancer or ductal carcinoma in situ (DCIS) diagnosed within 6 months of the screening date were defined as screen-detected cancers. Other women were followed up until next screening date or for 24 months whichever came first; for simplicity called 24 months. Women with a negative screening test and breast cancer/DCIS diagnosed within 24 months after the screening date or with a positive screening test and diagnosed with breast cancer/DCIS within 7–24 months after the screening date were defined as interval cancers. Women with screen-detected cancers and women with interval cancers together constituted the truly sick women. Women with a positive screening test and no diagnosis of breast cancer/DCIS were defined as false positive; and women with a negative screening test and no breast cancer/DCIS were defined as truly negative. The two latter groups together constituted the truly healthy women.

### Analysis

First, we calculated sensitivity (= screen detected/truly sick) and specificity (= truly negative/truly healthy) for Reader 1 both overall and by BI-RADS density code as set by Reader 1. We compared with the outcome of the consensus reading for the same group of women. In this calculation, the extra screen-detected cases in the consensus reading were considered overlooked by Reader 1 and therefore added as interval cancers for Reader 1, and the extra interval cancers in the consensus reading in women originally deemed positive by Reader 1 but reclassified as negative in the consensus reading were added as screen-detected cancers for Reader 1, Table 1. We calculated also the number of women with interval cancers and the number of women with a false-positive screening test per 1000 screened women.

**Table 1:** Number of screen detected and interval cancer in the Capital Region of Denmark 2012–2013 by reader (Reader 1, Reader 2, and Consensus) and by BI-RADS density code (as assesses by Reader 1, Reader 2, and in the Consensus reading)

Truly sick in Consensus reading	Truly sick in Reader 1 reading						Truly sick in Reader 2 reading					
	By Reader 1 BI-RADS code			By Consensus BI-RADS code			By Reader 2 BI-RADS code			By Consensus BI-RADS code		
	SDC	IC	Total	SDC	IC	Total	SDC	IC	Total	SDC	IC	Total
All BI-RADS												
SDC	370	46	416	370	46	416	347	69	416	347	69	416
IC	9	153	162	9	153	162	9	153	162	9	153	162
Total	379	199	578	379	199	578	356	222	578	356	222	578
BI-RADS 1												
SDC	101	9	110	81	7	88	92	19	111	70	18	88
IC	1	32	33	1	24	25	1	31	32	1	24	25
Total	102	41	143	82	31	113	93	50	143	71	42	113
BI-RADS 2												
SDC	170	22	192	162	22	184	164	28	192	159	25	184
IC	6	55	61	5	56	61	5	63	68	4	57	61
Total	176	77	253	167	78	245	169	91	260	163	82	245
BI-RADS 3												
SDC	90	13	103	112	15	127	85	18	103	105	22	127
IC	1	53	54	2	55	57	2	45	47	3	54	57
Total	91	66	157	114	70	184	87	63	150	108	76	184
BI-RADS 4												
SDC	9	2	11	15	2	17	6	4	10	13	4	17
IC	1	13	14	1	18	19	1	14	15	1	18	19
Total	10	15	25	16	20	36	7	18	25	14	22	36

*SDC* Screen detected cancer, *IC* Interval cancer

Second, we calculated the same measures for Reader 2 both overall and by BI-RADS density code as set by Reader 2. Third, we calculated the four measures for Reader 1, Reader 2, and for the consensus reading now using the consensus BI-RADS density code. The purpose of the first and second analyses was to measure the consequences of using one reader only as compared with the current consensus reading. The purpose of the third analysis was to measure the consequences of using one reader only in the hypothetical situation where the BI-RADS density code could be assessed automatically calibrated to the usual consensus level. 95% confidence interval for sensitivity and specificity are “exact” Clopper-Pearson confidence intervals [4]. Working under the assumption of independence between the readers, *p* values for difference in sensitivity and specificity were calculated using McNemar’s exact test. Statistical analyses were carried out with SAS 9.4. All plots were done in R 3.2.1, with ggplot2 and gridExtra packages.

## Results

There were 54,808 women in the study population. The majority of the mammography examinations, 69%, were read by radiologists who for different mammography examinations had acted both as first and second reader, and 31% of the mammography examinations were read by radiologists who had acted only as either first or second reader in the program. Reader 1 coded the mammography examinations from 3.5% of the women as positive; while this was the case for 3.0% of the women for Reader 2; and 3.1% in the consensus coding. Reader 1 found cancers in 0.68% of the women; while Reader 2 found cancers in 0.63% of the women. Consensus coding increased this percentage to 0.78%. Reader 1 had more women with false-positive outcome, 2.85%, than Reader 2, 2.36%, and the consensus code resulted in 2.35%.

Reader 1 coded 34% of the mammography examinations with BI-RADS density code 1, Table 2, and this proportion was the same for Reader 2, 35%, Table 3. There was, however, a considerable inconsistency in the density coding between the two readers, as both readers agreed on BI-RADS density code 1 for only 28% of the mammography examinations, Table 4. The proportion of mammography examinations with BI-RADS density code 2 ended up being almost the same for the three reader outcomes; 39%; 39%, and 40%, respectively. The proportions of mammography examinations with BI-RADS density codes 3 and 4 were as expected higher for the consensus outcome than for each of the individual readers. For BI-RADS density code 3 the proportions were 23%; 22%; and 27%, respectively. For BI-RADS density code 4, 4%; 3%; 5.0%, respectively, Tables 1, 2, and 3.

**Table 2:** Sensitivity and specificity of screening mammography in the Capital Region of Denmark 2012–2013 by Reader 1 and Consensus reading, stratified by BI-RADS density code as assessed by Reader 1

	Truly sick		Truly healthy		Total	%	Sensitivity (95% CI)	Specificity (95% CI)	Per 1000 screened	
	Positive^a^	Negative^b^	Positive^c^	Negative^d^					FN (IC)	FP
All BI-RADS										
Reader 1	379	199	1560	52,670	54,808	100.0	65.6 (61.5–69.4)	97.1 (97.0–97.3)	3.6 (3.1–4.2)	28.5 (27.1–29.9)
Consensus	416	162	1288	52,942	54,808	100.0	72.0 (68.1–75.6)	97.6 (97.5–97.7)	3.0 (2.5–3.4)	23.5 (22.2–24.8)
BI-RADS 1										
Reader 1	102	41	304	18,219	18,666	34.1	71.3 (63.2–78.6)	98.4 (98.2–98.5)	2.2 (1.6–3.0)	16.3 (14.5–18.2)
Consensus	110	33	277	18,246	18,666	34.1	76.9 (69.1–83.5)	98.5 (98.3–98.7)	1.8 (1.2–2.5)	14.8 (13.2–16.7)
BI-RADS 2										
Reader 1	176	77	756	20,534	21,543	39.3	69.6 (63.5–75.2)	96.4 (96.2–96.7)	3.6 (2.8–4.5)	35.1 (32.7–37.6)
Consensus	192	61	619	20,671	21,543	39.3	75.9 (70.1–81.0)	97.1 (96.9–97.3)	2.8 (2.2–3.6)	28.7 (26.5–31.1)
BI-RADS 3										
Reader 1	91	66	451	12,031	12,639	23.1	58.0 (49.8–65.8)	96.4 (96.0–96.7)	5.2 (4.0–6.6)	35.7 (32.5–39.1)
Consensus	103	54	349	12,133	12,639	23.1	65.6 (57.6–73.0)	97.2 (96.9–97.5)	4.3 (3.2–5.6)	27.6 (24.8–30.6)
BI-RADS 4										
Reader 1	10	15	49	1886	1960	3.6	40.0 (21.1–61.3)	97.5 (96.7–98.1)	7.7 (4.3–12.6)	25.0 (18.6–32.9)
Consensus	11	14	43	1892	1960	3.6	44.0 (24.4–65.1)	97.8 (97.0–98.4)	7.1 (3.9–12.0)	21.9 (15.9–29.4)

*SDC* Screen detected cancer, *IC* Interval cancer, *Cancer* SDC OR IC

^a^Defined as ResultX = Pos AND (SDC = 1 OR IC)

^b^Defined as ResultX = Neg AND (SDC = 1 OR IC)

^c^Defined as ResultX = Pos AND Cancer = 0

^d^Defined as ResultX = Neg AND Cancer = 0

**Table 3:** Sensitivity and specificity of screening mammography in the Capital Region of Denmark 2012–2013 by Reader 2 and Consensus reading, stratified by BI-RADS density code as assessed by Reader 2

	Truly sick		Truly healthy		Total	%	Sensitivity (95% CI)	Specificity (95% CI)	Per 1000 screened	
	Positive^a^	Negative^b^	Positive^c^	Negative^d^					FN (IC)	FP
All BI-RADS										
Reader 2	356	222	1291	52,939	54,808	100.0	61.6 (57.5–65.6)	97.6 (97.5–97.7)	4.1 (3.5–4.6)	23.6 (22.3–24.9)
Consensus	416	162	1288	52,942	54,808	100.0	72.0 (68.1–75.6)	97.6 (97.5–97.7)	3.0 (2.5–3.4)	23.5 (22.2–24.8)
BI-RADS 1										
Reader 2	93	50	292	18,872	19,307	35.2	65.0 (56.6–72.8)	98.5 (98.3–98.6)	2.6 (1.9–3.4)	15.1 (13.4–16.9)
Consensus	111	32	298	18,866	19,307	35.2	77.6 (69.9–84.2)	98.4 (98.3–98.6)	1.7 (1.1–2.3)	15.4 (13.7–17.3)
BI-RADS 2										
Reader 2	169	91	610	20,749	21,619	39.4	65.0 (58.9–70.8)	97.1 (96.9–97.4)	4.2 (3.4–5.2)	28.2 (26.0–30.5)
Consensus	192	68	619	20,740	21,619	39.4	73.8 (68.1–79.1)	97.1 (96.9–97.3)	3.1 (2.4–4.0)	28.6 (26.4–30.9)
BI-RADS 3										
Reader 2	87	63	350	11,665	12,165	22.2	58.0 (49.7–66.0)	97.1 (96.8–97.4)	5.2 (4.0–6.6)	28.8 (25.9–31.9)
Consensus	103	47	332	11,683	12,165	22.2	68.7 (60.6–76.0)	97.2 (96.9–97.5)	3.9 (2.8–5.1)	27.3 (24.5–30.3)
BI-RADS 4										
Reader 2	7	18	39	1653	1717	3.1	28.0 (12.1–49.4)	97.7 (96.9–98.4)	10.5 (6.2–16.5)	22.7 (16.2–30.9)
Consensus	10	15	39	1653	1717	3.1	40.0 (21.1–61.3)	97.7 (96.9–98.4)	8.7 (4.9–14.4)	22.7 (16.2–30.9)

*SDC* Screen detected cancer, *IC* Interval cancer, *Cancer* SDC OR IC

^a^Defined as ResultX = Pos AND (SDC = 1 OR IC)

^b^Defined as ResultX = Neg AND (SDC = 1 OR IC)

^c^Defined as ResultX = Pos AND Cancer = 0

^d^Defined as ResultX = Neg AND Cancer = 0

**Table 4:** Sensitivity and specificity of screening mammography in the Capital Region of Denmark 2012–2013 by reader stratified by BI-RADS density code as assessed in the consensus reading

	Truly sick		Truly healthy		Total	%	Sensitivity (95% CI)	Specificity (95% CI)	Per 1000 screened	
	Positive^a^	Negative^b^	Positive^c^	Negative^d^					FN (IC)	FP
All BI-RADS										
Reader 1	379	199	1560	52,670	54,808	100.0	65.6 (61.5–69.4)	97.1 (97.0–97.3)	3.6 (3.1–4.2)	28.5 (27.1–29.9)
Reader 2	356	222	1291	52,939	54,808	100.0	61.6 (57.5–65.6)	97.6 (97.5–97.7)	4.1 (3.5–4.6)	23.6 (22.3–24.9)
Consensus	416	162	1288	52,942	54,808	100.0	72.0 (68.1–75.6)	97.6 (97.5–97.7)	3.0 (2.5–3.4)	23.5 (22.2–24.8)
BI-RADS 1										
Reader 1	82	31	213	15,261	15,587	28.4	72.6 (63.4–80.5)	98.6 (98.4–98.8)	2.0 (1.4–2.8)	13.7 (11.9–15.6)
Reader 2	71	42	201	15,273	15,587	28.4	62.8 (53.2–71.7)	98.7 (98.5–98.9)	2.7 (1.9–3.6)	12.9 (11.2–14.8)
Consensus	88	25	202	15,272	15,587	28.4	77.9 (69.1–85.1)	98.7 (98.5–98.9)	1.6 (1.0–2.4)	13.0 (11.2–14.9)
BI-RADS 2										
Reader 1	167	78	738	20,690	21,673	39.5	68.2 (61.9–73.9)	96.6 (96.3–96.8)	3.6 (2.8–4.5)	34.1 (31.7–36.6)
Reader 2	163	82	594	20,834	21,673	39.5	66.5 (60.2–72.4)	97.2 (97.0–97.4)	3.8 (3.0–4.7)	27.4 (25.3–29.7)
Consensus	184	61	606	20,822	21,673	39.5	75.1 (69.2–80.4)	97.2 (96.9–97.4)	2.8 (2.2–3.6)	28.0 (25.8–30.2)
BI-RADS 3										
Reader 1	114	70	527	14,076	14,787	27.0	62.0 (54.5–69.0)	96.4 (96.1–96.7)	4.7 (3.7–6.0)	35.6 (32.7–38.8)
Reader 2	108	76	432	14,171	14,787	27.0	58.7 (51.2–65.9)	97.0 (96.7–97.3)	5.1 (4.1–6.4)	29.2 (26.6–32.1)
Consensus	127	57	414	14,189	14,787	27.0	69.0 (61.8–75.6)	97.2 (96.9–97.4)	3.9 (2.9–5.0)	28.0 (25.4–30.8)
BI-RADS 4										
Reader 1	16	20	82	2643	2761	5.0	44.4 (27.9–61.9)	97.0 (96.3–97.6)	7.2 (4.4–11.2)	29.7 (23.7–36.7)
Reader 2	14	22	64	2661	2761	5.0	38.9 (23.1–56.5)	97.5 (97.0–98.2)	8.0 (5.0–12.0)	23.2 (17.9–29.5)
Consensus	17	19	66	2659	2761	5.0	47.2 (30.4–64.5)	97.6 (96.9–98.1)	6.9 (4.1–10.7)	23.9 (18.5–30.3)

*SDC* Screen detected cancer, *IC* Interval cancer, *Cancer* SDC OR IC

^a^Defined as ResultX = Pos AND (SDC = 1 OR IC)

^b^Defined as ResultX = Neg AND (SDC = 1 OR IC)

^c^Defined as ResultX = Pos AND Cancer = 0

^d^Defined as ResultX = Neg AND Cancer = 0

The overall sensitivity for the consensus outcome was 72.0% and the specificity was 97.6%. Per 1000 screened women, 3.0 women experienced an interval cancer and 23.5 women had a false-positive screening test, Table 4. Reader 1 had an overall lower sensitivity of 65.6% (*p* < 0.0001) and a somewhat lower specificity of 97.1% (*p* < 0.0001). Reader 2 had an overall sensitivity of 61.6%(*p* < 0.0001), and the same specificity of 97.6% (*p* = 0.9498) as in the consensus reading, Tables 2 and 3.

**Fig. 1:**
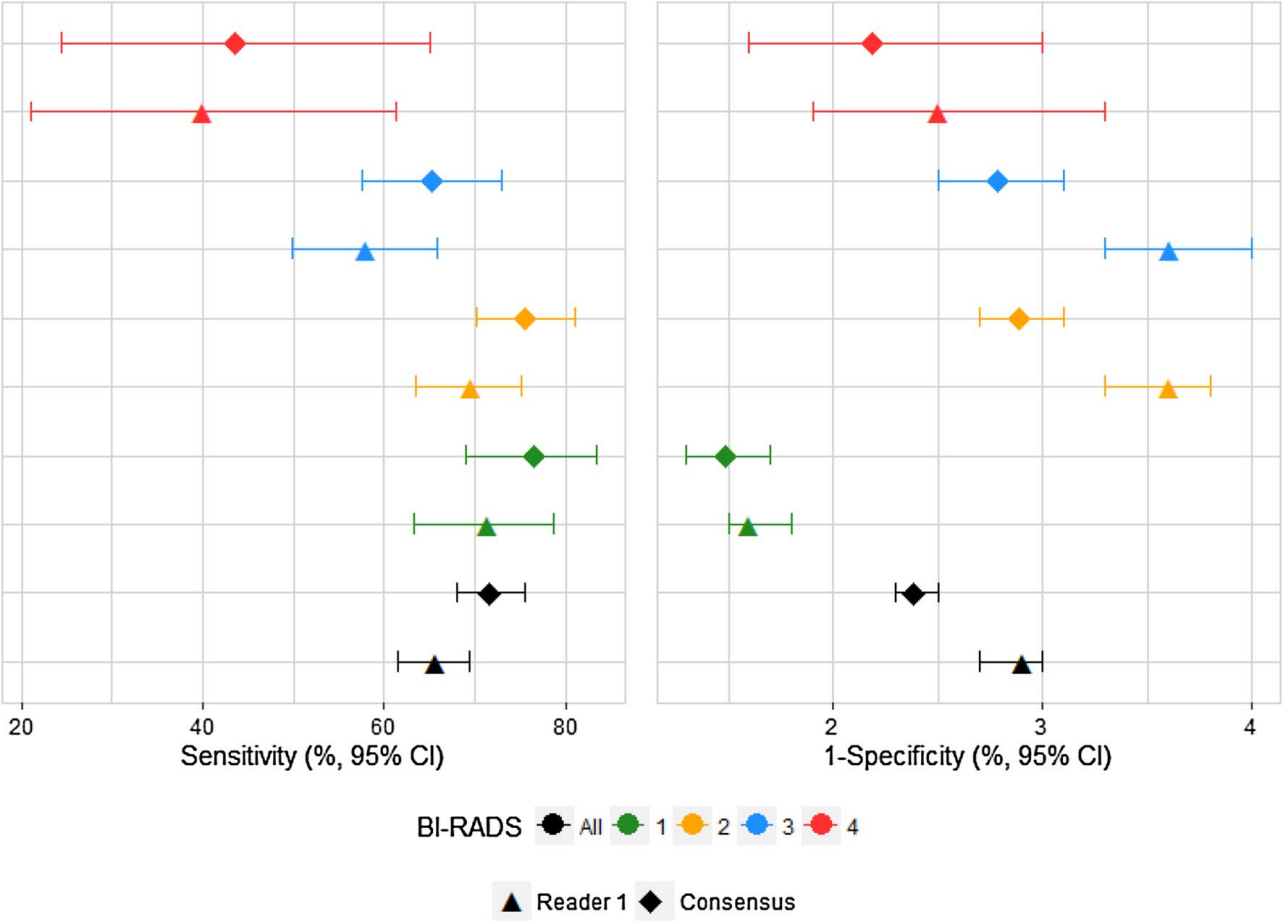
Sensitivity and specificity of screening mammography for Reader 1 and Consensus, by Reader 1 BI-RADS density code

**Fig. 2:**
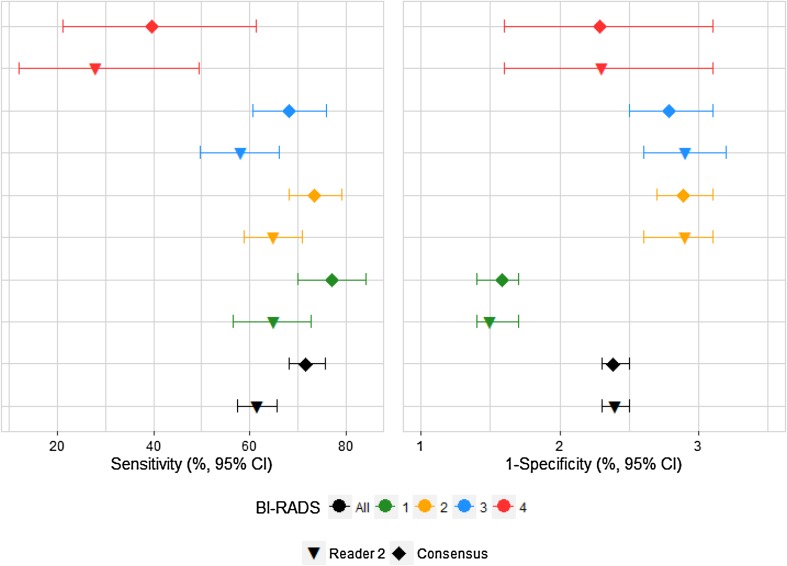
Sensitivity and specificity of screening mammography for Reader 2 and Consensus, by Reader 2 BI-RADS density code

When the mammography examinations were divided into the BI-RADS density groups set by Reader 1, both the sensitivity and the specificity for Reader 1 was lower than in the current consensus reading, e.g., for the 18,666 mammography examinations that Reader 1 coded as BI-RADS density code 1, Reader 1 had a sensitivity of 71.3% as compared with 76.9% in the consensus coding (*p* = 0.0215), Table 2 and Fig. 1. When the mammography examinations are divided into the BI-RADS density groups set by Reader 2, the sensitivity for Reader 2 was lower than in the current consensus reading, and the specificity remained at the same level, e.g., for the 19,307 mammography examinations that Reader 2 coded as BI-RADS density code 1, Reader 2 had a sensitivity of 65.0% as compared with 77.6% in the consensus coding (*p* < 0.0001), Table 3 and Fig. 2.

**Fig. 3:**
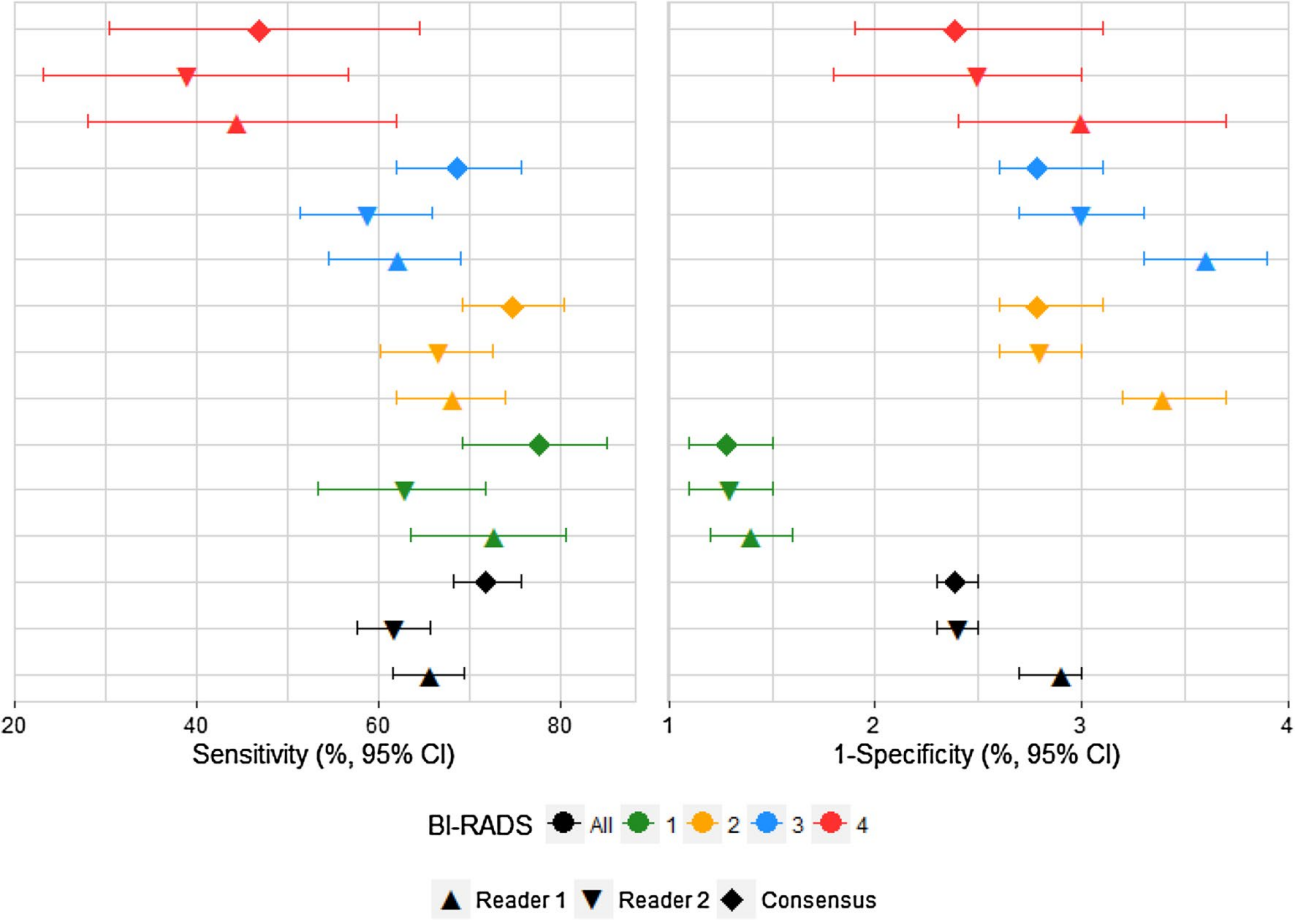
Sensitivity and specificity of screening mammography for Reader 1, Reader 2 and Consensus, by Consensus BI-RADS density code

When the mammography examinations were divided into the BI-RADS density groups set at the consensus reading both Reader 1 and Reader 2 had lower sensitivity for all BI-RADS density groups than found at the consensus reading. It should be noted though that for the 15,587 women with consensus BI-RADS density code 1; where Reader 1 had a sensitivity of 72.6%; Reader 2 of 62.8%, and the consensus reading of 77.9%, Table 4 and Fig. 3, there was no statistically significant difference in sensitivity between Reader 1 and the consensus reading (*p* = 0.0703), neither difference in specificity (*p* = 0.3824). For Reader 2 the sensitivity was statistically significantly lower than for consensus reading (*p* < 0.0001). For the small group of 2761 women with BI-RADS density code 4, both Reader 1 and Reader 2 had a sensitivity in line with that of the consensus reading (*p* = 1.000 and *p* = 0.3750, respectively).

## Discussion

### Main findings

The present days’ practice in screening mammography with consensus after double reading resulted in a sensitivity of 72.0% and a specificity of 97.6%. The highest sensitivity of 77.9% was amongst women in the BI-RADS density code 1 and the lowest of 47.2% amongst women in the BI-RADS 4 density code. The specificity was fairly consistent, between 98.7% and 97.2%. Per 1000 screened women this translated into 3 women with interval cancers and 24 women with a false-positive screening test. Our study showed a loss in sensitivity, although not always statistically significant, across all BI-RADS density groups if double reading was replaced by single reading. This was true both in the situations where we used the BI-RADS density codes set by one of the two readers, and in the situation where we used the BI-RADS density codes set in the consensus reading. For BI-RADS density code 1, the difference in sensitivity was not statistically significant between Reader 1 and consensus reading when the density code was set in the consensus reading, and both single readers had a specificity in agreement with the consensus reading. For BI-RADS density codes 2–3 there was a loss in specificity if Reader 1 was the single reader, but this was not the case if Reader 2 was the single reader.

### Other studies

In a number of case-control studies, Boyd et al. [5] found odds ratios of about 4 for the risk of breast cancer when women with more than 75% density were compared with women with less than 10% density. Our data, which included the screen-detected and the interval cancer cases, showed a doubling of the odds from BI-RADS density code 1 to BI-RADS density code 4; from 7 to 14 cases per 1000 screened women. In this perspective it seems reasonable to concentrate scarce screening resources on the high risk women. However, independent double reading of mammography examinations is recommended as standard practice in screening programs [1]. This is justified by the overall higher sensitivity of double as compared to single reading [2]. Furthermore, the ability of screening mammography to detect breast cancer decreases with increasing breast density. This has been shown both for radiologist assessed density [6], and more recently for automatically measured volumetric mammographic density [7].

The 34–35% of women with BI-RADS density code 1 found in the Danish program is high in an international perspective. In almost 4 million screening mammography examinations interpreted by radiologists who participate in the US Breast Cancer Surveillance Consortium (BCSC), only about 12% had BI-RADS density code 1, it should though be taken into account that screening in the US started normally at the age of 40 years [8]. A study from New York of women about the age of 50 years reported a proportion of 10% with BI-RADS density code 1 [9]. Similarly, in the German data reported by Weigel et al. [10], only 6% had BI-RADS density code 1. In data from the Norwegian breast cancer screening program, the distribution from BI-RADS 1 to 4 was 16%, 56%, 24%, and 4% [11]. In data from Malmö, Sweden, the distribution was 16%, 41%, 35%, and 8% [12].

Weigel et al. [10] reported data from 25,579 women screened age 50–69 years. The data came from a single screening unit in Germany, where abnormal findings detected by one or both readers resulted in mandatory consensus meeting of the two readers with a third.

Using the highest case reading, the overall sensitivity was 80.0%; 83.1% for mammography examinations with BI-RADS density code 2; 80.7% for BI-RADS density code 3; and 100% and 50%, respectively, for the small proportions of mammography examinations with either BI-RADS density code 1 or 4. It was not possible from the published data for calculate sensitivity by BI-RADS density code for single readers. To our knowledge no study previous to our’s has addressed the comprehensive impact of the reading schedule and breast density.

Reader 1 is normally the junior reader. It could therefore seem surprising that Reader 1 had a systematic, although statistically borderline non-significant, higher sensitivity than Reader 2, (*p* = 0.0505) This is, however, in agreement with the results of studies comparing radiographer and radiologist reading. In the UK National Health Service Breast Screening Program, screening units with radiographers had the same cancer detection rate as screening units with radiologists [13]. The recall rate was, however, higher in the units with radiographers than in the units with radiologists. In our data, Reader 1 has a statistically significant lower specificity than Reader 2, (*p* < 0.0001). This could indicate that the most difficult task in reading of mammograms is to avoid overcall.

### Strength and weaknesses

Our data derived from a population-based screening program. During the study period, the coverage of examination of targeted women was 73% [14]. Follow-up was complete because all diagnoses of breast cancer and DCIS are recorded in the Danish Pathology Register, and linkage to this register is possible based on the unique personal identification numbers. However, despite having a large data set, only 3–4% of the mammography examinations were coded with BI-RADS density code 4 by the individual readers. This meant that we had relatively few breast cancer cases in this high density group. The conclusions should be seen with reservations for wide and overlapping confidence intervals.

## Conclusion

Our study showed a loss in sensitivity - and to a lesser extent in specificity – meaning that the current double reading cannot be replaced by single reading without negative consequences for the screened women. This is true even if the BI-RADS density code could be set automatically calibrated to the usual consensus level. In the latter case, single reading could in some situation depending on the reader eventually be considered for women with BI-RADS density code 1.

## Data Availability

The dataset will be stored in the Danish Data Archive [15]  from with data can be accessed following the rules in the Danish legislation.
